# Interleukin-1β Drives Cellular Senescence of Rat Astrocytes Induced by Oligomerized Amyloid β Peptide and Oxidative Stress

**DOI:** 10.3389/fneur.2020.00929

**Published:** 2020-08-27

**Authors:** Dongsheng Shang, Yin Hong, Wangwang Xie, Zhigang Tu, Jun Xu

**Affiliations:** ^1^Institute of Life Sciences, Jiangsu University, Zhenjiang, China; ^2^China National Clinical Research Center for Neurological Diseases (NCRC-ND), Beijing Tiantan Hospital, Capital Medical University, Beijing, China; ^3^Department of Neurology, Beijing Tiantan Hospital, Capital Medical University, Beijing, China

**Keywords:** Alzheimer's disease, neuroinflammation, interleukin-1β, senescence, astrocyte, tau, amyloid β

## Abstract

**Background:** Alzheimer's disease (AD) is the leading cause of dementia. With no reliable treatment that delays or reverses the progress of AD, effective medical drugs, and interventions for AD treatment are in urgent need. Clinical success for patients thus relies on gaining a clearer understanding of AD pathogenesis to feed the development of novel and potent therapy strategies. It is well-established that inflammatory processes are involved in the pathology of AD, and recent studies implicated senescence of glial cells as an important player in the progression of AD.

**Methods:** We did a preliminary screen in rat astrocytes for the five most abundant inflammatory factors in neuroinflammation, namely IL-1β, IL-6, IL-8, TGF-β1, and TNF-α, and found that IL-1β could efficiently induce cellular senescence. After that, SA-β-gal staining, immunofluorescence, ELISA, qRT-PCR, and immunoblotting were used to explore the underlying mechanism through which IL-1β mediates cellular senescence of rat astrocytes.

**Results:** IL-1β-induced cellular senescence of rat astrocytes was accompanied by increased total and phosphorylated tau. Further experiments showed that both oligomerized amyloid β (Aβ) and H_2_O_2_ treatment can induce cellular senescence in rat astrocytes and increase the production and secretion of IL-1β from these cells. Subsequent mechanistic study revealed that activation of NLRP3 mediates Aβ and H_2_O_2_-induced maturation and secretion of IL-1β.

**Conclusion:** Our results suggest that IL-1β mediates senescence in rat astrocytes induced by several common adverse stimuli in AD, implicating IL-1β and NLRP3 as valuable diagnostic biomarkers and therapeutic targets for AD.

## Introduction

There are about 50 million people in the world living with Alzheimer's or other forms of dementia (1). As the leading cause of dementia, Alzheimer's disease (AD) is a progressive neurodegenerative disorder predominantly affecting people 65 years and older. The pathological features of AD include deposition of amyloid β peptide (Aβ), neurofibrillary tangles, and neuronal degeneration. Currently, for AD treatment, there are only a few medicines (most of them are cholinesterase inhibitors) for symptomatic treatment. But so far, there is no available medical treatment that delays or reverses the clinical courses of the disease, with novel and effective therapy strategies for AD urgently needed.

The vast majority of AD cases are sporadic, with unclear etiology. Nevertheless, it is widely accepted that the occurrence and development of sporadic AD is associated with various forms of brain insults over the years. Unfortunately, the sources of adverse stimuli can be diverse and extensive, including increased oxidative stress, protein misfolding, disturbances in calcium homeostasis, and energy deficiency, etc. (2). Recently, Ehsan et al. have showed that only one night of sleep deprivation results in a significant increase in Aβ burden in the right hippocampus and thalamus in human brains (3).

It is also well-accepted that inflammatory processes are involved in the pathology of AD (4). Elevated levels of inflammatory cytokines including interleukin (IL)-1β, IL-6, IL-8, tumor necrosis factor (TNF)-α, and transforming growth factor (TGF)-β1 are found in the brains of AD patients and animal models (5, 6). Most studies support that inflammation can promote the occurrence and development of AD, and inhibiting inflammation might help to prevent or alleviate AD (7, 8).

Cellular senescence is a state of cell growth arrest, often induced by various cellular stresses including oncogene activation, DNA damage, and telomere attrition (9, 10). Senescent cells are characterized by a flattened and enlarged morphology, increased senescence-associated β-galactosidase (SA-β-Gal) activity, and activated p16/pRB or p53/p21 pathways (11–14). In addition, senescent cells typically show a senescence-associated secretory phenotype (SASP) and secrete various pro-inflammatory cytokines (15, 16). We and the others have revealed that SASP factors can trigger senescence in surrounding cells and amplify senescence phenotypes (17–19). Interestingly, there is a significant overlap of secreted cytokines observed in AD and SASP (20).

Cellular senescence is associated with aging and is thus implicated as a potential cause of age-related neurodegenerative diseases (21, 22). Recently, Bussian et al. (23) implicated senescent cells in the etiology of AD in a P301S tauopathy mouse model, whereby senescent glial cells seemed to play a role in the initiation and progression of tau-mediated neurodegenerative diseases. About the same time, Musi et al. (24) reported a strong correlation between the presence of neurofibrillary tangles and cellular senescence in the brains of FTD associated P301L tauopathy mouse models. These papers suggest that cellular senescence plays an important role in the progression of tau-mediated neurodegenerative diseases.

As the most abundant cell type in the brain (25, 26), astrocytes control homeostasis and provide neuroprotection for the CNS (27, 28). Under physiological conditions, astrocytes supply neurons with energy, support synapses, regulate neurotransmitter levels, and release neurotrophic factors. In addition, the recent studies have shown that the astrocyte networks are essential for complex cerebral functions, such as sensation, cognition, and behavior (29, 30). According to the classical theories, astrocytes mainly respond to brain insults through a process called astrogliosis (31, 32), which usually protects but sometimes impairs the functions of the neural system when there is serious damage. Dysfunction in astrocytes was associated with the occurrence and development of AD (33, 34). Therefore, senescence of astrocytes might accelerate the AD process due to the loss of important cell functions in neurotrophic support and Aβ degradation (35–38).

Astrocytes can enter a senescent-like state *in vitro* after treatment with various stimuli (39, 40). Given that inflammatory factors can induce cellular senescence in several cell types (41, 42); the question arises as to whether inflammatory factors in the brain could induce senescence of astrocytes? To address this question, we did a preliminary screen in rat astrocytes for the five most abundant inflammatory factors in neuroinflammation, namely IL-1β, IL-6, IL-8, TGF-β1, and TNF-α, and found that IL-1β efficiently induced cellular senescence. We then further explored the roles of IL-1β in inducing astrocyte senescence and investigated the related mechanism.

## Materials and Methods

### Antibodies and Other Reagents

The antibodies used in the current study are listed in Supplementary Table 1. The other reagents used are as follows: recombinant rat IL-1β, IL-6, IL-8, and TNF-α (PeproTech, Rocky Hill, NJ, USA); recombinant rat transforming growth factor beta-1 (MyBioSource, San Diego, CA, USA); hydrogen peroxide solution, X-Gal (5-bromo-4-chloro-3-indolyl-β-D-galactopyranoside), and DAPI (4′, 6-diamidine-2′-phenylindole dihydrochloride) (Sigma-Aldrich, St. Louis, MO, USA); Trizol and Aβ (1–42) (Camarillo, CA, USA); HiScript II One Step qRT-PCR Probe Kit (Vazyme Biotechnology, Nanjing, China); and an interleukin-1β enzyme-linked immunosorbent assay kit (Beyotime Biotechnology, Shanghai, China). Aβ oligomer was prepared using the method described in the previous study (43).

### Primary Culture of Rat Astrocytes

All procedures involving rats were approved by the Jiangsu University Institutional Animal Care and Use Committee. P2-P3 neonatal SD rats were decapitated and the heads were placed into 70% alcohol for 5 min. Then, the nervous tissue, meningeal layer, brainstem, and cerebellum were removed, and the forebrains were trypsinized for 5 min at 37°C, centrifuged at 300 g for 5 min, and then cultured in DMEM supplemented with 10% fetal bovine serum (FBS) and 100 U/ml penicillin/100 μg/ml streptomycin (Life Technologies, Grand Island, NY, USA). Cells were incubated at 37°C in an atmosphere of 5% CO_2_ and 5% O_2_. After 5 days, purified astrocytes were obtained through the purification step (44). Briefly, the mixed cells were shaken at 200 rpm overnight at 37°C. After the supernatant was removed, the remaining cells were cultured with fresh medium.

### SA-β-Gal Staining

SA-β-Gal staining was performed as previously described (45–47). Briefly, treated cells were fixed in 2% formaldehyde/0.2% glutaraldehyde in PBS for 5 min at room temperature. After that the slides were incubated in the staining solution (40 mM Na_2_HPO_4_, 150 mM NaCl, 2 mM MgCl_2_, 5 mM K_3_Fe (CN) _6_, 5 mM K_4_Fe(CN)_6_, 1 mg/mL X-gal, pH 6.0) for 10 h at 37°C. Images were captured using an upright microscope (Nikon Eclipse, Tokyo, Japan).

### Senescence-Associated Heterochromatic Foci (SAHF) Staining

The formation of SAHF is one of the most important cellular senescence markers. The method of SAHF staining was adapted from a previous study (48). Cells were fixed with 4% formaldehyde in PBS for 10 min and permeablized with 0.2% Triton X-100 for 5 min at room temperature. After that the slides were incubated with 1 μg/μL of DAPI for 5 min at room temperature and sealed. Images were obtained using the fluorescence microscope (Nikon Eclipse). Positive cells (>5 foci per cell) were counted in five different fields of each slide.

### Immunofluorescence (IF) Staining

IF staining was performed as described previously (49, 50). In brief, cells were fixed in 4% paraformaldehyde in PBS for 10 min at room temperature. Fixed cells were permeablized with 0.2% of Triton X-100 for 5 min and blocked with 3% BSA in PBS for 30 min at room temperature. After that cells were incubated with a selected primary antibody with an appropriate dilution for 2 h at room temperature, and then incubated with an appropriate fluorescent secondary antibody for 1 h at room temperature. Finally, the slides were stained with 1 μg/μL of DAPI for 5 min and sealed with neutral balsam. The antibodies used are listed in Supplementary Table 1. Images were captured using Nikon Eclipse.

### Treatment of Cells

We plated 3 × 10^4^ cells into the wells of 24-well plates with coverslips, and 3 × 10^5^ cells into 6-cm dishes. Cells were treated with medium containing various concentrations of inflammatory factors, H_2_O_2_, or Aβ for 2 days. The treated cells on coverslips were used for SA-β-gal, SAHF, and IF staining. The treated cells in 6-cm dishes were used for ELISA, qRT-PCR, and Immunoblotting.

### Tomato Lectin Staining

Tomato lectin staining was used to identify microglial cells (51, 52). Cells were fixed in 4% paraformaldehyde in PBS for 10 min at room temperature, permeabilized with 0.2% of Triton X-100 for 5 min, and blocked with 3% bovine serum albumin (BSA) in PBS for 30 min at room temperature. Then, cells were incubated with Tomato lectin-FITC (1:500) for 2 h at room temperature and stained for 5 min with 1 μg/μL DAPI. Images were captured as above.

### Enzyme-Linked Immunosorbent Assay (ELISA)

The supernatant of cells treated with or without Aβ (300 ng/mL) or H_2_O_2_ (30 μM) for 2 days were collected and used to quantify the concentration of IL-1β by ELISA, according to the manufacturer's instructions.

### qRT-PCR and Immunoblotting

qRT-PCR was carried out using the HiScript II One Step qRT-PCR Probe Kit, according to the manufacturer's instructions. The primer sequences used in this study are listed in Supplementary Table 2. The endogenous control was β-actin. Immunoblotting was performed using the antibodies listed in Supplementary Table 1, and as described in previous studies (45, 46). β-actin was used as an internal control.

### Statistical Analysis

Data are presented as mean ± SD unless otherwise noted and were analyzed for significance between groups using Student's *t*-test (two-tailed) or one-way analysis of variance (ANOVA) according to the need using GraphPad Prism version 7.00 (San Diego, CA, USA). *P* < 0.05 was considered statistically significant. ^#^*P* ≥ 0.05; ^*^P < 0.05; ^**^P < 0.01; ^***^P < 0.001.

## Results

### Primary Culture of Rat Astrocytes

After 5 days of primary culture, the mixed cells were identified using IF staining. Astrocytes, identified by their expression of GFAP (38, 53), comprised ~58% of the primary cultured cells (Supplementary Figure 1A). Similarly, tomato lectin staining (54, 55) revealed a microglial cell density of ~23%. After the purification step, the density of astrocytes increased to around 96% (Supplementary Figure 1B), ensuring our further research work.

### IL-1β Can Induce Cellular Senescence in Rat Astrocytes

Brain inflammation often involves expression of IL-1β, IL-6, IL-8, TGF-β1, and TNF-α, thus we started our screening experiments with these commercially available inflammatory factors. Increased β-galactosidase activity and formation of SAHF in cells were used as the major markers of cellular senescence (19). The concentrations of these inflammatory factors were arbitrarily set at 1, 3, and 10 ng/ml. As shown in Supplementary Figure 2, while the other factors had no obvious effects on cells, IL-1β significantly increased the β-galactosidase activity and formation of SAHF in cells in a dose-dependent manner when the concentration of IL-1β was not greater than 3 ng/ml. The maximal increases in the proportions of positive cells were 2.9 and 3.2 in the SA-β-gal and SAHF assays, respectively. These results suggested that IL-1β can induce cellular senescence in rat astrocytes.

**Figure 1:**
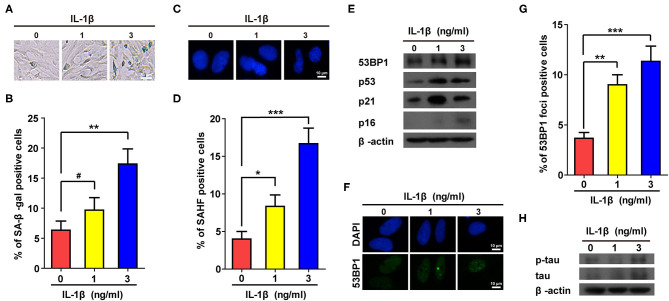
IL-1β treatment induced senescent phenotypes in rat astrocytes. **(A,C)** Representative pictures of SA-β-gal and DAPI staining of rat astrocytes treated with different concentrations (0, 1, and 3 ng/ml) of IL-1β for 2 days. **(B,D)** Statistical analysis of **(A,C)**. **(E)** Protein levels of 53BP1, p53, p21, and p16 in rat astrocytes treated with different concentrations of IL-1β. **(F)** Representative IF staining of 53BP1 in rat astrocytes treated with different concentrations of IL-1β for 2 days, the white scale bar indicates 10 μm. **(G)** Statistical analysis of **(F)**. **(H)** protein levels of total and phosphorylated tau in rat astrocytes treated with different concentrations of IL-1β. Data indicate the mean values calculated from three independent experiments (±SD).

To confirm the above results, IL-1β (1 and 3 ng/ml) was used to treat astrocytes. Consistent with Supplementary Figure 2, treatment using IL-1β dramatically increased the proportions of positive cells in both SA-β-gal and SAHF assays (Figures 1A–D). In addition, protein markers of senescence such as p53, p21, and p16, were also up-regulated in cells treated with IL-1β (Figure 1E). Cellular senescence is often accompanied by accumulation of DNA damage, which is often manifested by increased numbers of foci and protein level of 53BP1. As expected, IL-1β-treated cells showed increased values for both (Figures 1E–G), and more importantly, the IL-1β-induced cellular senescence of astrocytes was accompanied by the increased expression and phosphorylation of tau (Figure 1H).

### IL-1β Can Activate SASP in Rat Astrocytes

**Figure 2:**
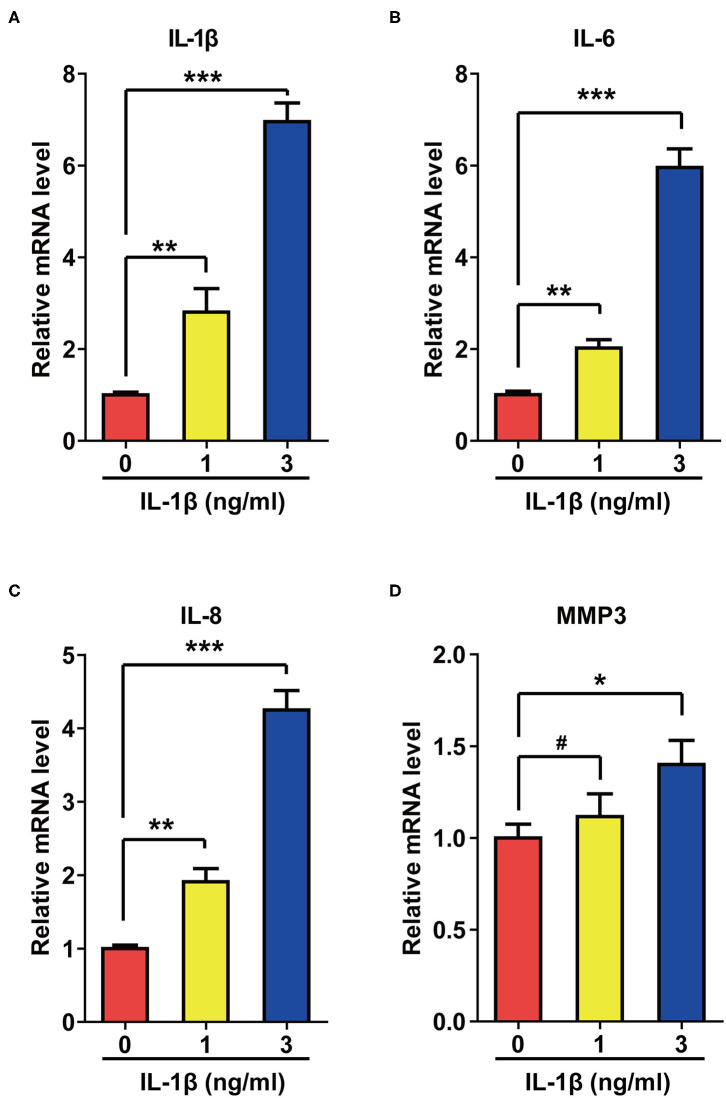
IL-1β treatment activated the SASP pathway in rat astrocytes. **(A–D)** mRNA levels of IL-1β **(A)**, IL-6 **(B)**, IL-8 **(C)**, and MMP3 **(D)** in rat astrocytes treated with different concentrations of IL-1β for 2 days. Data indicate the mean values calculated from three independent experiments (±SD).

Activation of SASP pathways is an important characteristic of cellular senescence. Since IL-1β can induce cellular senescence, we speculated that this cytokine might also activate SASP pathways. As expected, IL-1β at different concentrations increased the mRNA levels of IL-6, IL-8, Matrix Metallopeptidase 3 (MMP3), and IL-1β itself to different extents (Figure 2).

### Aβ Treatment Can Induce Cellular Senescence in Rat Astrocytes

**Figure 3:**
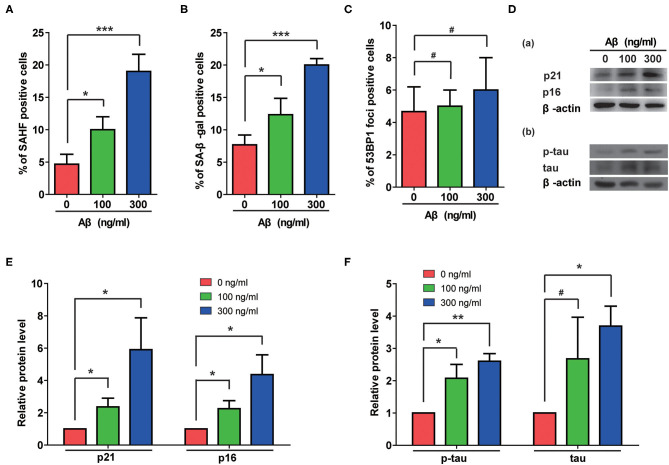
Aβ treatment induced senescent phenotypes and upregulation of total and phosphorylated tau protein in rat astrocytes. **(A–C)** Statistical analysis of SAHF- **(A)**, SA-β-gal- **(B)**, and 53BP1-foci- **(C)** positive cells in rat astrocytes treated with different concentrations of Aβ for 2 days. **(D)** Protein levels of p21 and p16 (a), p-tau and tau (b) in rat astrocytes following treatment with different concentrations of Aβ. **(E,F)** Quantification of **(D)**. Data indicate the mean values calculated from three independent experiments (±SD).

The Aβ accumulation that is common in AD cases is considered a possible pathogenic factor. We therefore assessed the effects of Aβ treatment on rat astrocytes. As shown in Figures 3A,B, Aβ treatment significantly increased the portions of β-gal- and SAHF-positive cells. Although no DNA damage accumulated in these cells (Figure 3C), the Aβ treatment indeed induced senescence in the astrocytes that was further confirmed by increased protein levels of p21 and p16 (Figure 3D-a). More importantly, Aβ treatment also induced the upregulation of total and phosphorylated-tau in rat astrocytes as the cells became senescent (Figure 3D-b). Furthermore, the quantification of immunoblotting results showed that Aβ treatment significantly increased the protein levels of p21, p16, p-tau, and tau in a dose-dependent manner (Figures 3E,F).

### H_2_O_2_ Treatment Can Induce Cellular Senescence in Rat Astrocytes

**Figure 4:**
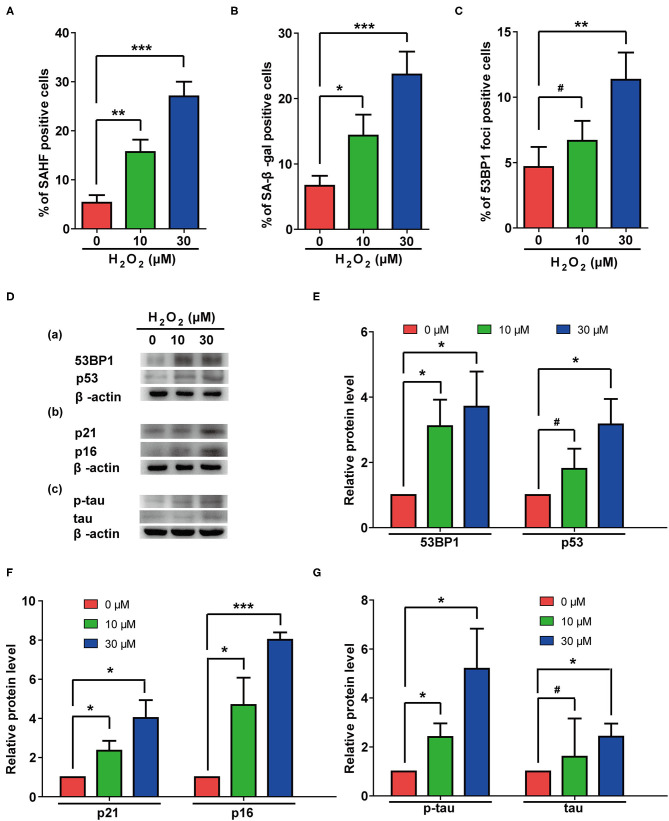
H_2_O_2_ treatment induced senescent phenotypes and upregulation of total and phosphorylated tau protein in rat astrocytes. **(A–C)** Statistical analysis of SAHF- **(A)**, SA-β-gal- **(B)**, and 53BP1-foci- **(C)** positive cells in rat astrocytes treated with different concentrations of H_2_O_2_ for 2 days. **(D)** Protein levels of 53BP1 and p53 (a), p21 and p16 (b), p-tau and tau (c) in rat astrocytes after treated with different concentrations of H_2_O_2_. **(E–G)** quantification of **(D)**. Data indicate the mean values calculated from three independent experiments (±SD).

Oxidative pressure is closely related to the occurrence of AD, and H_2_O_2_ treatment is often used to simulate increased oxidative pressure on cells. In our study, H_2_O_2_ treatment induced cellular senescence manifested by increased rates of β-gal- and SAHF-positive cells (Figures 4A,B). Additionally, H_2_O_2_ treatment significantly increased numbers of 53BP1 foci (Figure 4C) and the protein levels of p53 and 53BP1 in cells (Figures 4D-a, E). These results suggested that H_2_O_2_ treatment caused excessive DNA damage in these cells. The elevated protein levels of p21 and p16 (Figures 4D-b, F) further confirmed that H_2_O_2_ treatment can cause cellular senescence in rat astrocytes. Interestingly, H_2_O_2_ treatment also upregulated total and phosphorylated-tau in these cells (Figures 4D-c, G).

### Both Aβ and H_2_O_2_ Treatment Can Activate SASP Pathways and Induce Secretion of IL-1β From Rat Astrocytes

**Figure 5:**
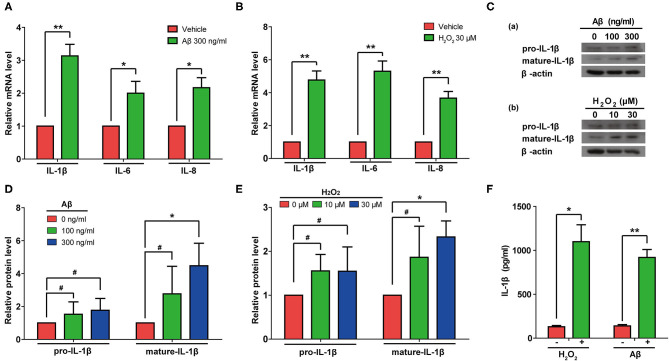
Aβ and H_2_O_2_ treatment both activated the SASP pathway and induced secretion of IL-1β in rat astrocytes. **(A,B)** mRNA levels of IL-1β, IL-6, and IL-8 in rat astrocytes treated with or without Aβ **(A)** or H_2_O_2_ **(B)** for 2 days. **(C)** Protein levels of pro-IL-1β and mature-IL-1β in rat astrocytes treated with different concentrations of Aβ (a) or H_2_O_2_ (b) for 2 days. **(D,E)** Quantification of **(C)**. **(F)** ELISA quantification of the concentration of IL-1β in the supernatant of rat astrocytes treated with or without Aβ (300 ng/mL) or H_2_O_2_ (30 μM) for 2 days. Data indicate the mean values calculated from three independent experiments (±SD).

Next, we measured the mRNA levels of IL-1β, IL-6, and IL-8 in rat astrocytes treated with Aβ or H_2_O_2_. As expected, the mRNA levels of IL-1β, IL-6, and IL-8 all increased in cells treated with either Aβ or H_2_O_2_ (Figures 5A,B). Consistently, immunoblotting results confirmed that both Aβ and H_2_O_2_ treatment increased the protein levels of mature IL-1β in cells (Figures 5C–E). Of note, although the protein levels of pro-IL-1β increased mildly, these changes did not reach a significant level in our experiments (Figures 5D,E). These results indicated that both Aβ and H_2_O_2_ treatment can activate SASP pathways. In addition, we found increased IL-1β protein levels in conditioned media from the astrocyte cultures (Figure 5F) after both the Aβ and H_2_O_2_ treatments. Importantly, the levels of IL-1β reached ~1 ng/ml, a concentration previously shown to induce cellular senescence.

### Aβ and H_2_O_2_ Treatment Both Can Activate NLRP3 in Rat Astrocytes

**Figure 6:**
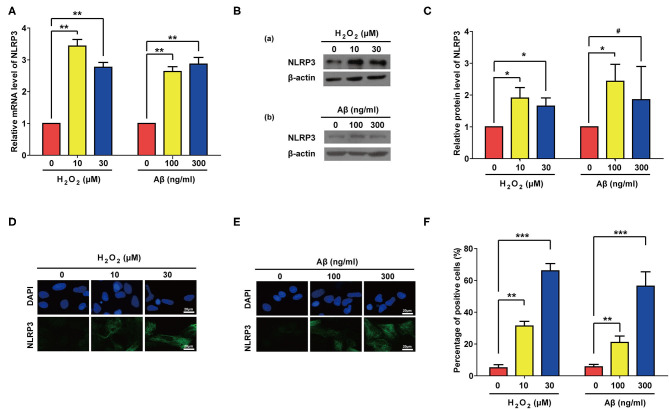
Both Aβ and H_2_O_2_ treatment activated NLRP3 pathway in rat astrocytes. NLRP3 mRNA levels **(A)** and protein levels **(B)** in rat astrocytes treated with different concentrations of H_2_O_2_ or Aβ for 2 days. **(C)** Quantification of **(B)**. **(D,E)** Representative IF staining of NLRP3 in the treated cells, the white scale bar indicates 20 μm. **(F)** statistical analysis of **(D,E)**. Data indicate the mean values calculated from three independent experiments (±SD).

Previous studies have showed that NLR Family Pyrin Domain Containing 3 (NLRP3) senses cellular stresses, activates IL-1β transcription and promotes its maturation and secretion (56–58). We therefore accessed the expression levels of NLRP3 after Aβ or H_2_O_2_ treatment. As expected, both Aβ and H_2_O_2_ treatment significantly increased the mRNA and proteins levels of NLRP3 in our experiments (Figures 6A–C). Of note, compared to the control group, the increase of the protein level of NLRP3 in the group treated with 300 ng/ml of Aβ did not reach a significant level. This may be due to the large standard deviation (Figure 6C). Nevertheless, IF results confirmed the significant increase of NLRP3 expression in rat astrocytes treated with Aβ or H_2_O_2_ (Figures 6D–F). These data demonstrate that both Aβ and H_2_O_2_ treatment can increase mRNA and protein levels of NLRP3 in rat astrocytes.

## Discussion

It has long been speculated that cellular senescence is closely related to the occurrence and development of AD (59). Although *in vitro* evidence has been reported from time to time (60–62), the necessary *in vivo* evidence only began to accumulate recently (23, 24, 63).

In this study, we showed for the first time that the inflammatory factor IL-1β can induce cellular senescence in primary cultured rat astrocytes (Figures 1, 2). Moreover, we demonstrated that both Aβ stimulation and oxidative stress can also induce senescence in rat astrocytes, and this process is accompanied by increased synthesis and secretion of IL-1β (Figures 3–5). Therefore, we speculate that during the development of AD, multiple adverse stimuli cause the senescence of rat astrocytes, and then IL-1β transmits and amplifies this phenomenon. Indeed, previous studies may provide some support for this hypothesis. In one such example, Parajuli et al. (64) reported that Aβ induces IL-1β processing via the production of reactive oxygen species in microglia, however their findings could not exclude the possibility that this phenomenon is accompanied by cellular senescence in glial cells. Previous studies have shown that increased IL-1β secretion is often accompanied by activation of the NLRP3 pathway (65, 66). More specifically, Halle (67) showed that in response to Aβ, microglia secrete more IL-1β via activation of the NLRP3 pathway. Youm et al. (58) demonstrated that demonstrated that the canonical NLRP3 inflammasome links systemic low-grade inflammation to multiple age-related degenerative changes, such as thymic involution, reduced innate immune activation, and decreased brain function. Consistent with these previous findings, we also observed activation of the NLRP3 pathway in rat astrocytes treated by Aβ and H_2_O_2_, and NLRP3 activation (Figure 6), in turns, might increase the maturation of IL-1β. Our results are based on *in vitro* experiments only, *in vivo* experiments using appropriate animal models may be carried out in the future to further confirm the role of IL-1β in development of AD.

As mentioned previously, inflammation is a fundamental response to injuries in central nervous system and is associated with the occurrence and development of AD. Astrocytes were reported to secrete a broad profile of inflammatory factors (2). Consistently, our study showed that treatment with Aβ, H_2_O_2_, or IL-1β induced astrocytes to be senescent and to secrete several important inflammatory factors, such as IL-6 and IL-8. These results raise the following questions: In the brain, do inflammatory factors lead to cellular senescence; or does cellular senescence lead to the rise of inflammatory factors; or do these two processes crosstalk to each other as a positive feedback loop? With the existing evidence, currently we are unable to answer these questions and will pay close attention to any progress in this area.

Our recently published study (19) showed that IL-1β induces cellular senescence through EGFR activation. Interestingly, EGFR activation is common in astrogliosis (68, 69), while EGF treatment can trigger astrogliosis (70). Although it was not investigated in this study, we speculate that EGFR activation may play a role in cellular senescence of astrocytes induced by IL-1β. This raises the interesting question of whether there is some intrinsic relationship between senescence and astrogliosis in astrocytes. Due to the lack of evidence at this stage, we cannot make any judgment yet. But the question is very interesting, and worthy of further exploration.

Until now, there is no ideal animal models of sporadic AD since none of them perfectly simulates all aspects of the pathological process of AD (71). That's why many treatments and interventions had been successful in preclinical models but failed in clinical trials. Are we looking in the wrong direction? Therefore, research into novel mechanisms of AD etiology is urgently needed, and astrocyte senescence induced by inflammatory factors contributing to the occurrence and progress of AD may be a very promising research direction. In addition, searching for astrocyte-specific senescence markers as early diagnostic markers for AD is an attractive research goal.

## Conclusion

Overall, our current study, together with previous *in vivo* and clinical evidence (72–75), suggests that IL-1β and NLRP3 actively function in promoting senescence of astrocytes and could be valuable diagnosis biomarkers and therapeutic targets for AD.

## Data Availability Statement

All datasets generated for this study are included in the article/Supplementary Material.

## Ethics Statement

The animal studies were reviewed and approved by the Jiangsu University Institutional Animal Care and Use Committee.

## Author Contributions

JX, ZT, and YH conceived and designed the study. DS and WX performed experiments and acquired data. DS and YH performed data analysis. JX, ZT, and YH drafted the manuscript. JX and ZT obtained the fundings. All authors edited and revised the manuscript. All authors contributed to the article and approved the submitted version.

## Conflict of Interest

The authors declare that the research was conducted in the absence of any commercial or financial relationships that could be construed as a potential conflict of interest.

## Supplementary Material

Supplementary Figure 1 Primary culture of rat astrocytes. **(A)** The representative pictures of immunofluorescence staining of GFAP and tomato lectin on the mixed cells. **(B)** The representative pictures of immunofluorescence staining of GFAP on purified rat astrocytes. The mean values showed in the pictures calculated from three independent experiments.

Supplementary Figure 2 Inflammatory factor screening using SA-β-gal and SAHF assays. **(A,B)** Cells were treated with different concentrations of inflammatory factors for 2 days, and the percentages of SA-β-gal **(A)** and SAHF **(B)** positive cells were analyzed. Data indicate the mean values calculated from three independent experiments (±SD). Statistically significant differences were determined by one-way ANOVA (^**^*P* < 0.01, ^***^*P* < 0.001).

Supplementary Figure 3 The graphic abstract.

Supplementary Table 1 Antibodies used in this study.

Supplementary Table 2 Primers sequences used in this study.
