# Supplementary figures and images for: Interleukin-1β Drives Cellular Senescence of Rat Astrocytes Induced by Oligomerized Amyloid β Peptide and Oxidative Stress
Source: Front Neurol. 2020 Aug 27;11:929. doi: 10.3389/fneur.2020.00929 (PMC7493674; doi:10.3389/fneur.2020.00929)

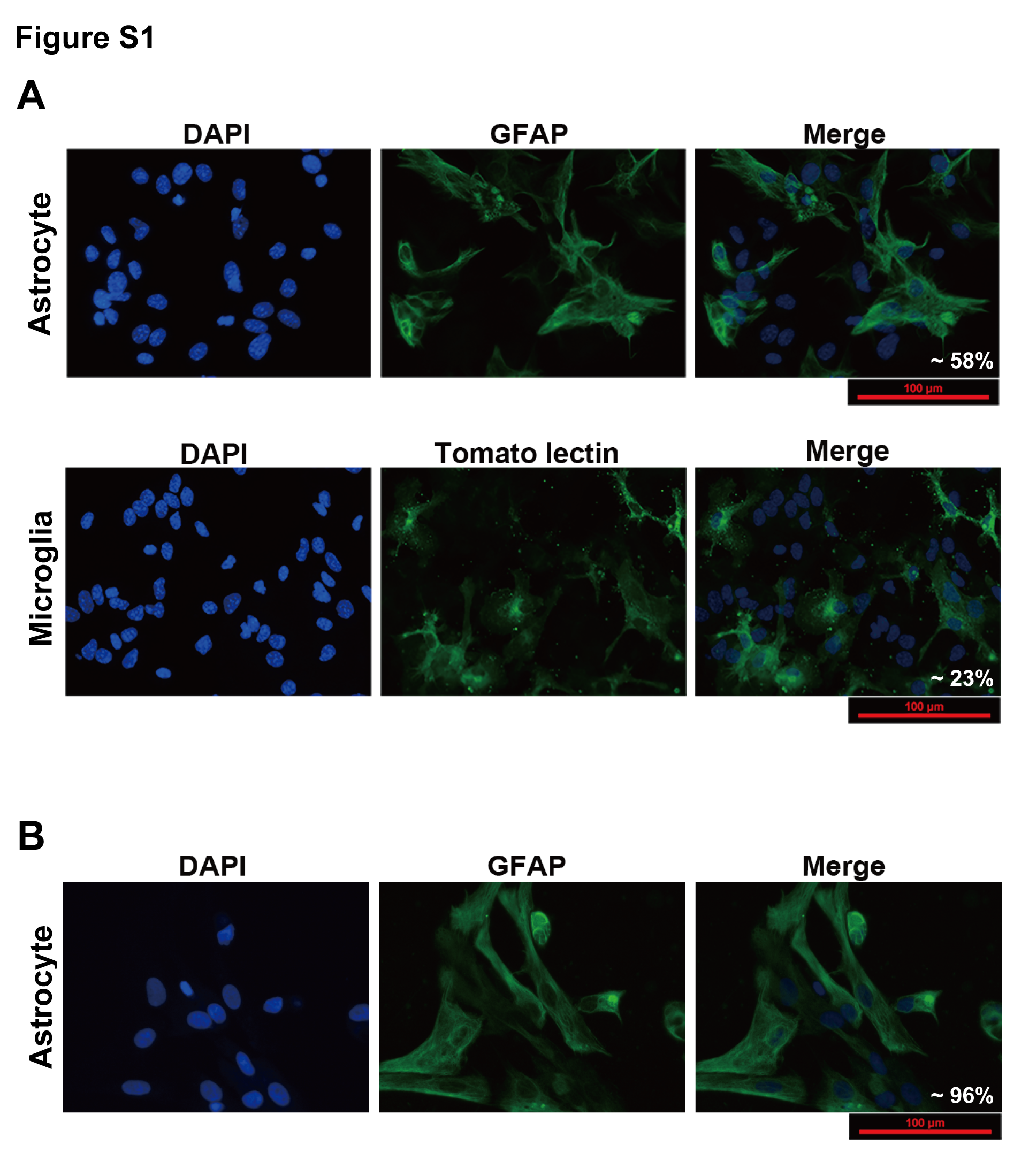

Supplement: Supplementary Figure 1 — Primary culture of rat astrocytes. (A) The representative pictures of immunofluorescence staining of GFAP and tomato lectin on the mixed cells. (B) The representative pictures of immunofluorescence staining of GFAP on purified rat astrocytes. The mean values showed in the pictures calculated from three independent experiments. [file Image_1.TIF]

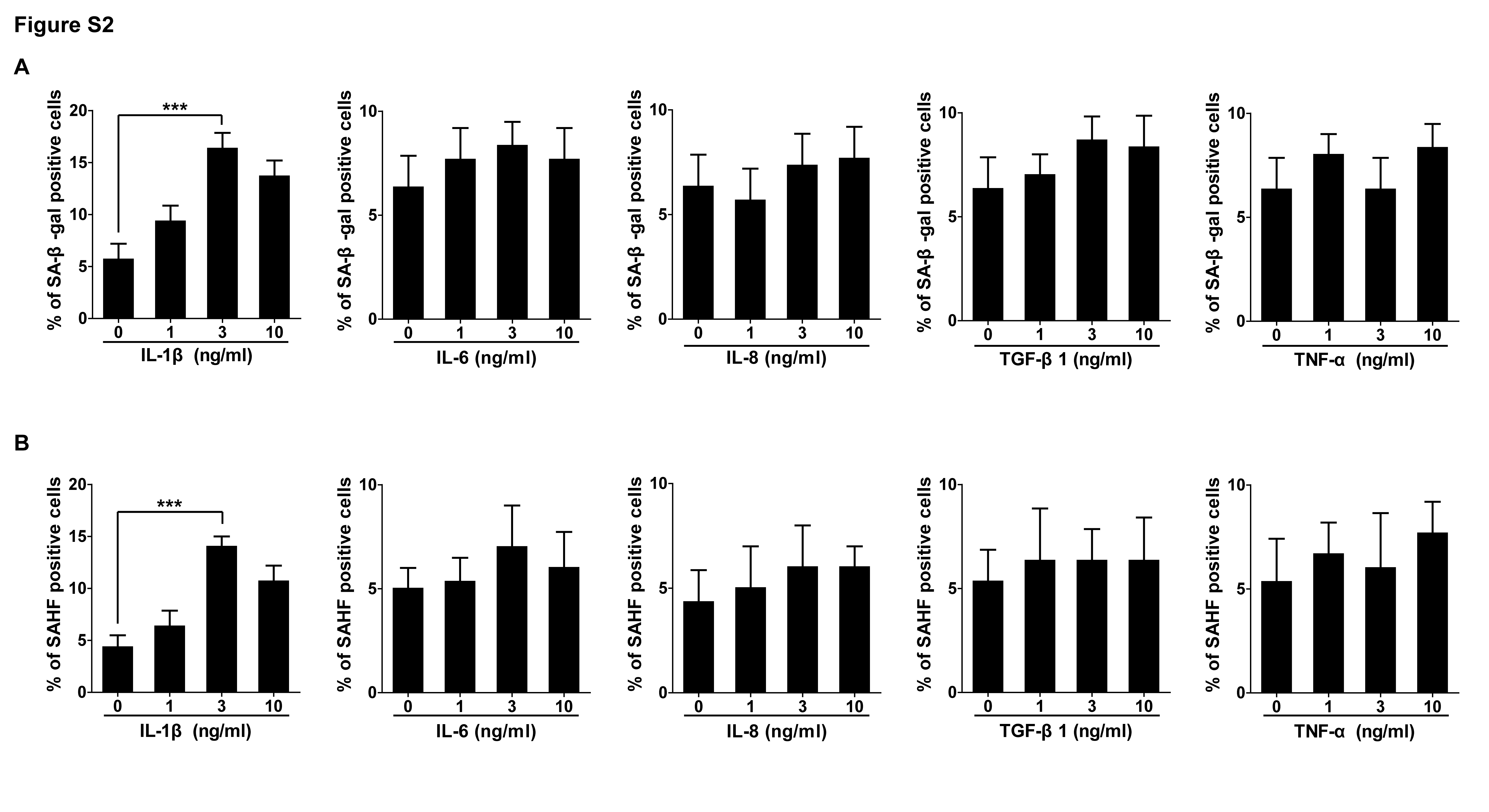

Supplement: Supplementary Figure 2 — Inflammatory factor screening using SA-β-gal and SAHF assays. (A,B) Cells were treated with different concentrations of inflammatory factors for 2 days, and the percentages of SA-β-gal (A) and SAHF (B) positive cells were analyzed. Data indicate the mean values calculated from three independent experiments (±SD). Statistically significant differences were determined by one-way ANOVA (**P < 0.01, ***P < 0.001). [file Image_2.TIF]

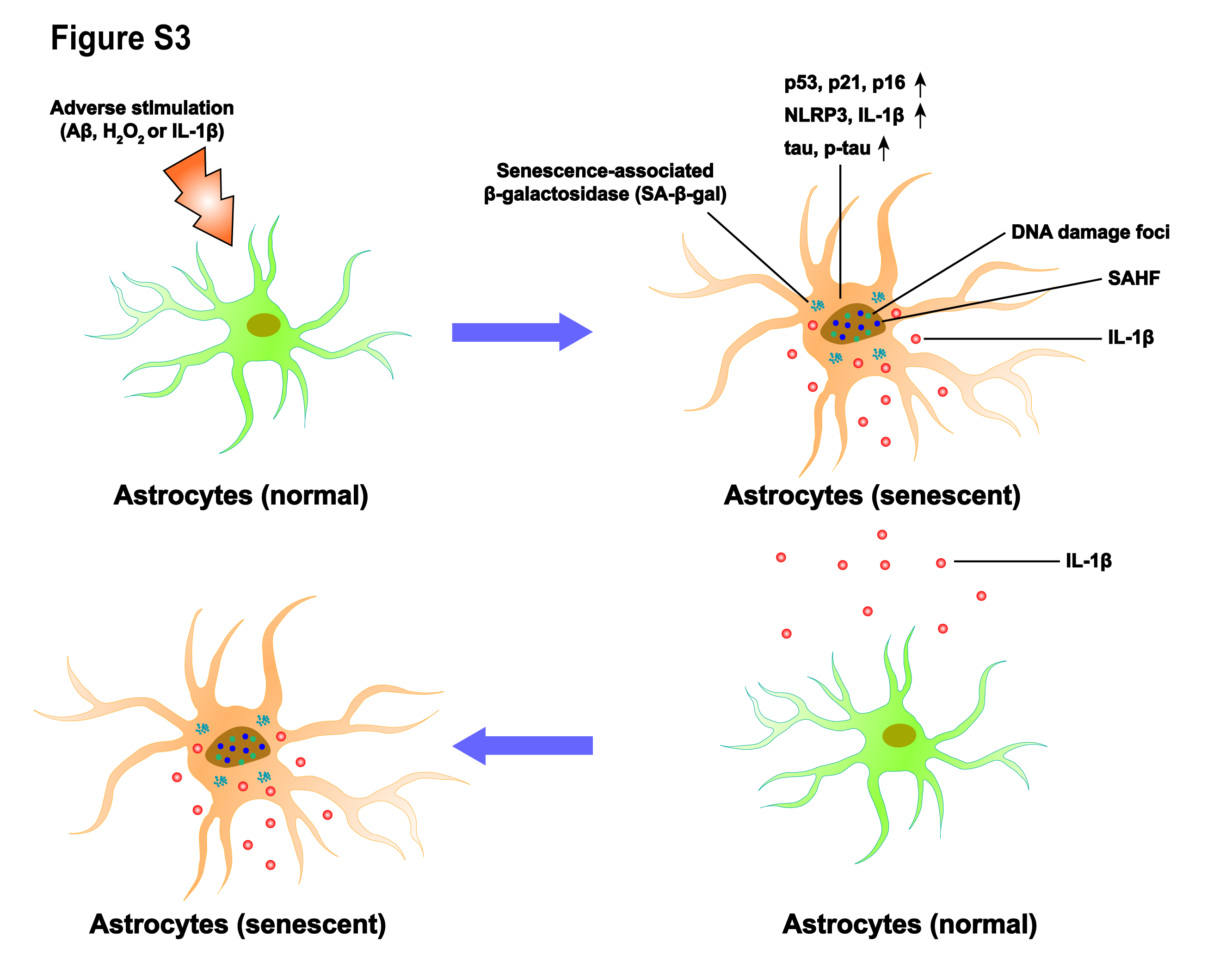

Supplement: Supplementary Figure 3 — The graphic abstract. [file Image_3.TIF]
