# Supplementary material for: Interleukin-1β Drives Cellular Senescence of Rat Astrocytes Induced by Oligomerized Amyloid β Peptide and Oxidative Stress
Source: Front Neurol. 2020 Aug 27;11:929. doi: 10.3389/fneur.2020.00929 (PMC7493674; doi:10.3389/fneur.2020.00929)
Supplement: Supplementary Table 1 — Antibodies used in this study. [file Table_1.DOC]

**Supplementary Table 1. Antibodies used in this study.**

| **Targeted protein** | **Source** | **Company** | **Application** | **Dilution ratio** |
| --- | --- | --- | --- | --- |
| 53BP1 | rabbit | BETHYL | Immunofluorescence & Western blot | 1:1000 |
| p53 | mouse | Millipore | Western blot | 1:1000 |
| p21 | rabbit | Santa Cruz | Western blot | 1:1000 |
| p16 | mouse | BD | Western blot | 1:1000 |
| tau | rabbit | Abcam | Western blot | 1:1000 |
| p-tau(Thr231) | mouse | Invitrogen | Western blot | 1:1000 |
| IL-1β | rabbit | Abcam | Western blot | 1:1000 |
| NLRP3 | rabbit | Cell Signaling Technology | Immunofluorescence & Western blot | 1:1000 |
| GFAP | rabbit | Abcam | Immunofluorescence | 1:1000 |
| Lectin from Lycopersicon esculentum (tomato)-FITC conjugate |  | Sigma | Immunofluorescence | 1:500 |
| β-actin | mouse | Sigma | Western blot | 1:5000 |
| Goat anti-rabbit Alexa-fluor 488 | goat | Life Technologies | Immunofluorescence | 1:1000 |
| Goat anti-Mouse IgG(H+L)HRP | goat | Jackson ImmunoResearch | Western blot | 1:10000 |
| Goat anti-Rabbit IgG(H+L)HRP | goat | Jackson ImmunoResearch | Western blot | 1:10000 |
