# Supplementary material for: Interleukin-1β Drives Cellular Senescence of Rat Astrocytes Induced by Oligomerized Amyloid β Peptide and Oxidative Stress
Source: Front Neurol. 2020 Aug 27;11:929. doi: 10.3389/fneur.2020.00929 (PMC7493674; doi:10.3389/fneur.2020.00929)
Supplement: Supplementary Table 2 — Primers sequences used in this study. [file Table_2.DOC]

Supplementary Table 2. Primers sequences used in this study.

| **Name** | **Sequences(5’to 3’)** |
| --- | --- |
| IL-1β | Ctctgtgactcgtgggatga |
|  | agattcttccccttgaggcc |
| IL-6 | Ccacccacaacagaccagta |
|  | actccagaagaccagagcag |
| IL-8 | Gaagatagattgcaccga |
|  | catagcctctcacacatttc |
| MMP3 | Gggctatccgaggtcatgaa |
|  | tgcctggaaagttctcagct |
| NLRP3 | Tctgcgtgttgtcaggatct |
|  | acagtgaagtaaggccggaa |
| β-actin | Tgtgttgtccctgtatgcct |
|  | aatgtcacgcacgatttccc |
